# Transcriptomics and weighted protein network analyses of the LRRK2 protein interactome reveal distinct molecular signatures for sporadic and LRRK2 Parkinson’s Disease

**DOI:** 10.1038/s41531-024-00761-8

**Published:** 2024-08-03

**Authors:** Yibo Zhao, Matthew Bracher-Smith, Yuelin Li, Kirsten Harvey, Valentina Escott-Price, Patrick A. Lewis, Claudia Manzoni

**Affiliations:** 1 UCL School of Pharmacy, dept Pharmacology, London, UK; 2 University of Cardiff, School of Medicine, Division of Psychological Medicine and Clinical Neurosciences, Cardiff, UK; 3 Dementia Research Institute, Cardiff University, Cardiff, UK; 4 Royal Veterinary College, London, UK; 5 UCL Queen Square Institute of Neurology, London, UK

**Keywords:** Systems biology, Parkinson's disease

## Abstract

Mutations in the *LRRK2* gene are the most common genetic cause of familial Parkinson’s Disease (LRRK2-PD) and an important risk factor for sporadic PD (sPD). Multiple clinical trials are ongoing to evaluate the benefits associated with the therapeutical reduction of LRRK2 kinase activity. In this study, we described the changes of transcriptomic profiles (whole blood mRNA levels) of LRRK2 protein interactors in sPD and LRRK2-PD cases as compared to healthy controls with the aim of comparing the two PD conditions. We went on to model the protein-protein interaction (PPI) network centred on LRRK2, which was weighted to reflect the transcriptomic changes on expression and co-expression levels of LRRK2 protein interactors. Our results showed that LRRK2 interactors present both similar and distinct alterations in expression levels and co-expression behaviours in the sPD and LRRK2-PD cases; suggesting that, albeit being classified as the same disease based on clinical features, LRRK2-PD and sPD display significant differences from a molecular perspective. Interestingly, the similar changes across the two PD conditions result in decreased connectivity within a topological cluster of the LRRK2 PPI network associated with protein metabolism/biosynthesis and ribosomal metabolism suggesting protein homoeostasis and ribosomal dynamics might be affected in both sporadic and familial PD in comparison with controls.

## Introduction

Leucine-rich repeat kinase 2 is a large (>250 kDa), multifunctional enzyme encoded by the *LRRK2* gene, possessing 2 enzymatic (GTPase and kinase) and 4 scaffold (armadillo, ankyrin, LRR and WD40 motifs) domains^1^. LRRK2 is able to interact with a large number of protein partners (Zhao et al. ^2^), and is involved in a range of biological processes including vesicular transport, autophagy, regulation of cellular response to stress, regulation of cell cycle, etc^3–6^. Mutations in the *LRRK2* gene are an important genetic cause of familial PD (fPD), with 1 to 40% of fPD cases associated with coding variants in LRRK2, depending on the population under study^7–10^. Since 2004, when the first variants in the *LRRK2* gene were associated with fPD, numerous coding and non-coding variants of LRRK2 have been identified in PD families. These include the G2019S and R1441C/G mutations, which are the 2 most common pathogenic variants occurring on the kinase and GTPase domains of the LRRK2 protein, leading to an increased kinase activity and decreased GTPase activity respectively^11–15^. Additionally, polymorphisms, mainly in the promoter of *LRRK2* and proposed to modulate expression of LRRK2, have been linked to lifetime risk of developing sporadic PD (sPD) (Nalls et al. ^16^), while upregulated LRRK2 kinase activity (in the absence of pathogenic mutations) has also been related with sPD.

The molecular mechanism(s) underlying the contribution of LRRK2 to both fPD and sPD are as yet unclear, despite the extensive efforts made to investigate LRRK2 function in health and disease. For example, PD-related inflammation both in the Central Nervous System (CNS) and at the periphery, was linked to an increased LRRK2 expression level and strengthened LRRK2 kinase activity in microglia and peripheral immune cells in sPD patients as compared to controls (Cook et al. ^17^; Di Maio et al. ^18^). These data indicate that LRRK2 is crucial for the understanding of PD etiopathogenesis, and that LRRK2 might constitute a link between familial and sporadic forms of the disease. There is extensive evidence suggesting that LRRK2 is a crucial regulator of the crosstalk between the CNS and the periphery, possibly via modulation of the immune system^19,20^. LRRK2 exhibits high expression levels in monocytes, and its protein levels are increased in peripheral immune cells in PD patients as compared to controls^21,22^. Some evidence suggests that the presence of the LRRK2-G2019S and the LRRK2-R1441G mutations in peripheral immune cells alone is sufficient for inflammation-induced dopaminergic neuronal loss in genotypically normal mouse brain, suggesting that the dysfunction in the peripheral immune system may be pivotal for the disease, at least in the fPD condition^23^. Our previous study found that a large number of LRRK2 protein interactors are characterised by a significantly higher expression in whole blood, as compared to their levels in the brain regions and other peripheral tissues such as liver, lung and kidney^2^, again suggesting the relevance of LRRK2 within the peripheral immune system.

A second observation is that, from a clinical perspective, LRRK2-PD and sPD have been reported to present with distinct features. Despite having similar motor-symptoms (such as bradykinesia, tremor, rigidity, and postural instability) as well as sharing some of the non-motor symptoms^24–26^, patients with LRRK2-PD display slower decline considering both movement and cognitive impairment^27,28^. In addition, LRRK2-PD and sPD show slightly different pathological features. For example, LRRK2-PD patients exhibit less α-synuclein aggregation in Cerebrospinal Fluid (CSF), feature that is a hallmark of sPD^29,30^ as well as increased basal forebrain volume, which is probably a compensation of the cholinergic system^31^. Such differences might highlight an intrinsic variation at the molecular level between these 2 forms of PD thus suggesting different model systems might be required to investigate them. Also, this consideration may pose a problem in translational research, for example the use of LRRK2 inhibitors in clinical trials^32^ might require patient stratification.

Based on the above observations, in this study, we hypothesised that despite important overlaps in their clinical presentation, sPD and LRRK2-PD might have a different molecular signature and therefore the molecular alterations contributing to disease onset and progression might be functionally different. We decided to focus on whole blood transcriptomics, based upon the relevance of LRRK2 as potential regulator of the immune activity. We constructed the protein-protein interaction (PPI) network around LRRK2 (LRRK2_net_) and evaluated the whole-blood mRNA expression changes within the LRRK2_net_ in a cohort of sPD and LRRK2-PD patients in comparison with healthy controls. The results provide a bioinformatic demonstration that the signature of expression changes in the LRRK2_net_ shows both similarities and differences in sPD vs LRRK2-PD. Of particular interest are the statistically significant differences in both gene expression and co-expression that suggest some of the molecular pathways at the base of these two conditions might be different. Our finding is relevant for the understanding of the different molecular mechanisms of PD, and it highlights the necessity for patient stratification in both discovery research and clinical trials, suggesting different therapeutic approaches might be needed if we intend to move from symptomatic to effective disease treatment.

## Results

### Whole blood transcriptomic profiling of the LRRK2 interactome

A total of 418 protein interactors of LRRK2 (LRRK2_int_) were retrieved via an in-house pipeline developed in our previous study^2^ (Table S1). Tissue-specific expression scores of 378 LRRK2 interactors in the whole blood were extracted from the same study. Among the 418 LRRK2 interactors, 140 (37.0%) presented significantly higher expression scores in blood when compared to other peripheral tissues (liver, lung and kidney) and brain regions (Figure S1). Functional enrichment analysis performed on this selection of LRRK2 interactors returned 234 GO-BP terms (Table S2). After semantic grouping of GO:BP terms, text cloud analysis of the enrichment results showed that terms in the groups of “response to stimulus”, “immune response” and “apoptosis” (*N* = 100/234, accounting for 42.7% of all enriched GO terms) contained keywords highly associated with immune functions, such as “cytokine”, “leucocyte”, “lymphocyte”, etc (Figure S1). Overall, these results suggested that a substantial proportion of LRRK2 protein interactors might be involved in the regulation of the immune functions at the periphery.

### Whole blood transcriptomic profiling of the LRRK2 interactome in the PPMI cohort

Whole blood RNA-Seq read counts were retrieved from the PPMI dataset for 415 (out of 418) LRRK2 interactors and for 657 subjects with validated genotyping data (controls = 170; sPD cases = 371; and LRRK2-PD cases = 116). A total of 38 interactors were removed due to low read counts. No subjects were identified as outliers by PCA (Figure S2). Hence, mRNA levels of the remaining 377 LRRK2 interactors of 657 PPMI subjects formed the PPMI_Matrix. Demographic features of the included cohorts are listed in Table 1 with no significant difference in sex and age nor in motor symptom severity when the sPD and LRRK2-PD cohorts were compared. Of note, 95.5% of the 3 cohorts have white ancestry. Among the LRRK2-PD cases, 100 (86.2%) were LRRK2-G2019S carriers while 16 (13.8%) were LRRK2-R1441C/G carriers. Therefore, and to avoid bias induced by different LRRK2 variants, LRRK2-R1441C/G carriers were removed, leaving a total of 641 PPMI subjects for further analysis.

**Table 1:** PPMI cohort characterisation

	Control (*N* = 170)	sPD (*N* = 371)	LRRK2-PD (*N* = 116)
Sex (Male)	94 (55.3%)	224 (60.4%)	71 (61.2%)
Age (Mean (SD))	60.9 (0.8)	61.3 (0.5)	63.1 (0.8)
Ethnicity (White)	160 (94.1%)	360 (97.0%)	108 (93.1%)
MDS-UPDRS III	–	21.1 (0.5)	20.1 (0.9)
LRRK2-G2019S carrier	–	–	100 (86.2%)
LRRK2-R1441C/G carrier	–	–	16 (13.8%)

Note: Movement disability for the sPD and LRRK2-PD cases was evaluated via MDS-UPDRS III (Score range: 0–132; 32 and below is mild, 59 and above is severe)^53,54^. T-test showed there was no significant difference in MDS-UPDRS III scores between the sPD and LRRK2-PD cases.

**Fig. 1:**
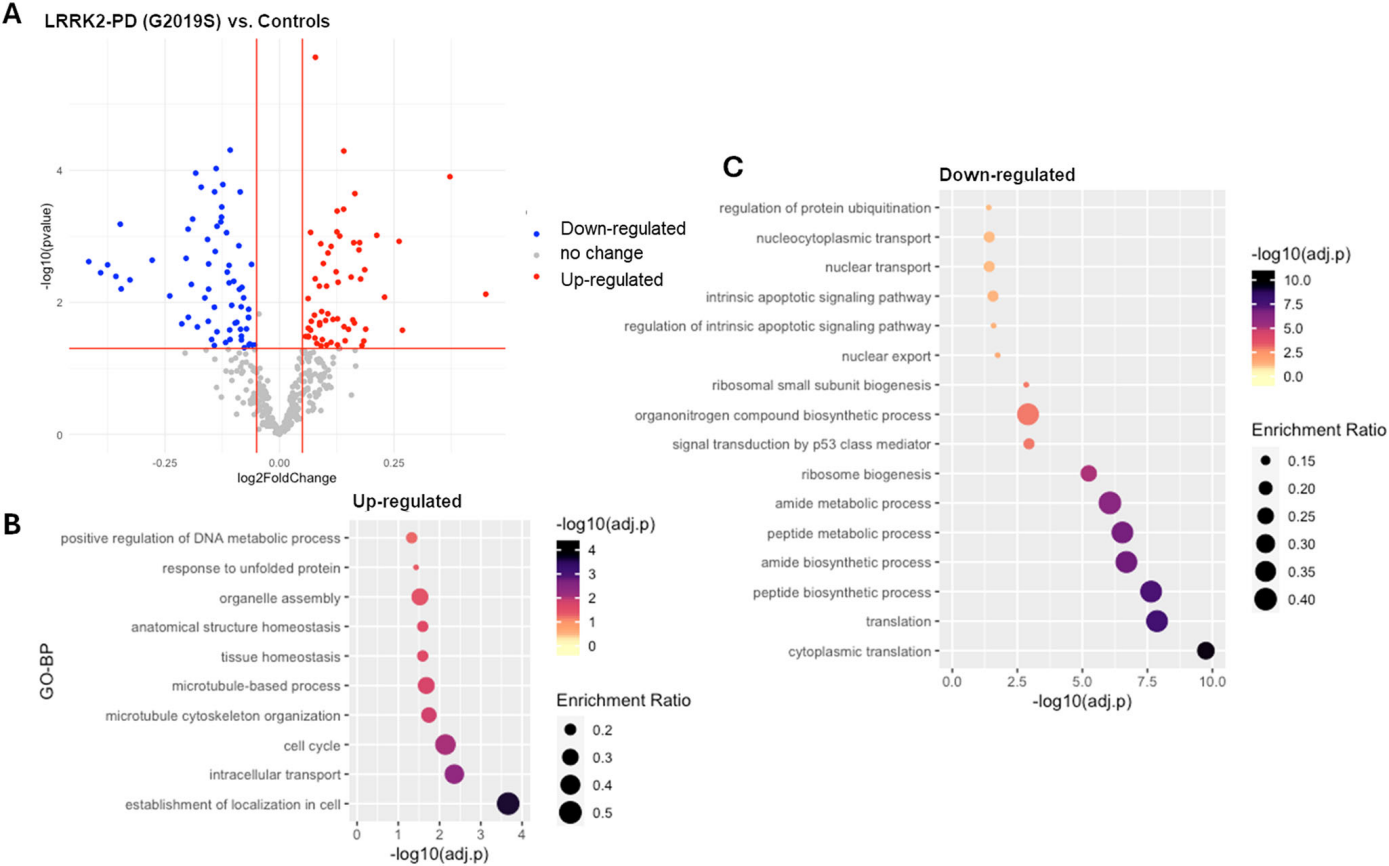
DEA on whole-blood mRNA levels of LRRK2 interactors in the LRRK2-PD cases vs. Controls. **A** The scatter plot shows results from DEA performed on LRRK2 interactors; LRRK2-PD (LRRK2-G2019S) cases vs. Controls. Interactors with significant alterations (|log2FC| > 0.05 & adjusted-*p* < 0.05) are colour coded as blue (down-regulated) and red (up-regulated) dots. **B**, **C** The bubble graphs show the enriched GO-BPs for up-regulated and down-regulated LRRK2 interactors; LRRK2-PD cases vs. Controls. The colour of the bubble represents enrichment significance (−log10(adjusted-*p*)), while bubble size represents enrichment ratio (intersection size/query size).

DEA results (Table S3) showed that: the mRNA levels of 67 interactors (17.7%) were significantly altered in the LRRK2-PD cases vs. controls, with 39 down-regulated and 28 up-regulated interactors (|log2(FC) > 0.05|, adjusted-*p* < 0.05, Fig. 1A, Table S3). Functional enrichment analysis related the up-regulated interactors mainly to cytoskeletal dynamics/transport, while the down-regulated interactors were mainly related to biosynthetic processes and ribosome biogenesis (Fig. 1B, C, Table S4).

**Fig. 2:**
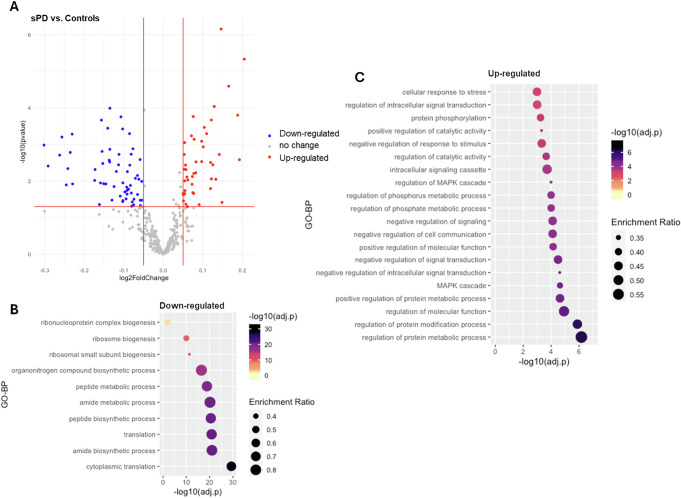
DEA on whole-blood mRNA levels of LRRK2 interactors in the sPD cases vs. Controls. **A** The scatter plot shows results from DEA performed on LRRK2 interactors; sPD cases vs. Controls. Interactors with significant alterations (|log2FC| > 0.05 and adjusted-*p* < 0.05) are colour coded as blue (down-regulated) and red (up-regulated) dots. **B**, **C** The bubble graphs show the enriched GO-BPs for up-regulated and down-regulated LRRK2 interactors; sPD cases vs. Controls. The colour of the bubble represents enrichment significance (−log10(adjusted-*p*)), while bubble size represents enrichment ratio (intersection size/query size). Of note, for up-regulated interactors, only top 20 GO-BPs are shown in the graph.

As for the sPD cases, a total of 55 interactors (14.6%) presented significant changes in expression levels in comparison with controls, including 28 down-regulated and 27 up-regulated interactors (|log2(FC) > 0.05|, adjusted-*p* < 0.05, Fig. 2A, Table S3). Functional enrichment analysis showed that the up-regulated interactors were associated with GO-BP terms related to protein metabolic processes and signalling, while the down-regulated interactors, similar to the LRRK2-PD condition, were predominantly related to biosynthetic processes and ribosome biogenesis (Fig. 2B, C, Table S4).

**Fig. 3:**
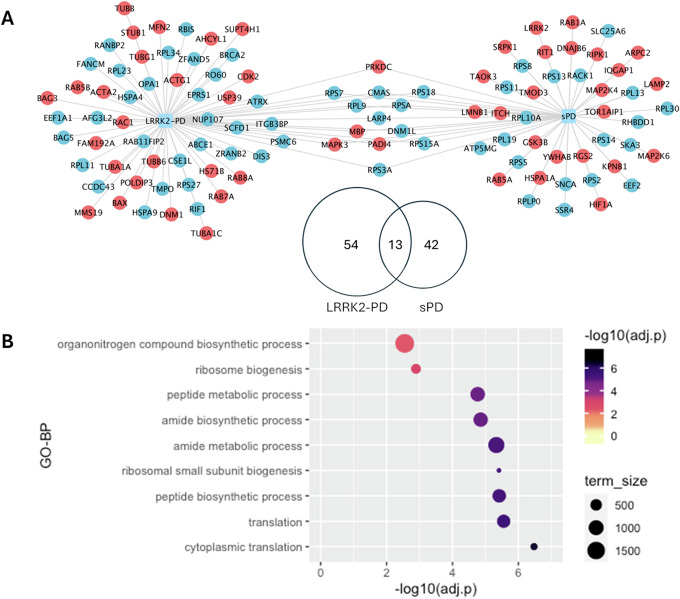
DEA on LRRK2 interactors in the LRRK2-PD and sPD cases vs. Controls. **A** The Venn diagram and the network graph show 13 LRRK2 interactors presenting the same differential expression pattern in the LRRK2-PD and the sPD cohorts in comparison with controls. In the network graph, interactors with significant differential expression profiles are colour-coded based on up-regulation (red) and down-regulation (blue). **B** The bubble graph shows the GO-BP terms enriched for the 13 LRRK2 interactors that presented similar alterations in the 2 PD conditions as compared to controls.

Of note, only a total of 13 interactors exhibited the same alteration in LRRK2-PD and sPD (Fig. 3A), in which 9 interactors were down-regulated while 4 interactors were up-regulated, suggesting these LRRK2 interactors were consistently affected during PD progression regardless of the existence of the *LRRK2-G2019S* mutation. Interestingly, 9 out of these 13 LRRK2 interactors were found in the interactome of other PD genes *SNCA, PRKN, PINK1, PARK7, VPS35, FBXO7*; Supplementary Data S1.) Functional enrichment analysis associated these 13 proteins with protein metabolism/biosynthesis and ribosomal metabolism (Fig. 3B).

### Transcriptomic profiles of the LRRK2 interactors differentiate LRRK2-PD and sPD cases

**Fig. 4:**
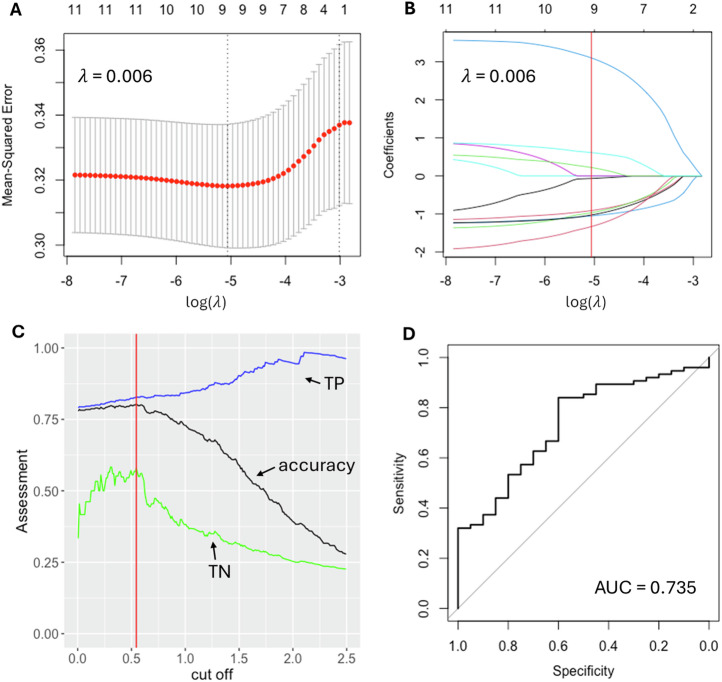
ML model for sPD/LRRK2-PD differentiation based on the transcriptomic profiles of the LRRK2_int_. **A** The logistic regression model with LASSO (Least Absolute Shrinkage and Selection Operator) was adopted to reduce dimensionality and select the most significant expression profiles for the LRRK2 interactors able to differentiate sPD and LRRK2-PD. λ value of 0.006, with log(λ) = −5.062 was selected according to 10-fold cross-validation. **B** LASSO coefficient profiles of 11 LRRK2 interactors are plotted. The optimal coefficient profile was produced against the selected λ (marked as the vertical red line). **C** The distribution curve shows different cut-off values and the model performance (as assessed by accuracy) on the train set. A cut-off of 0.54 was selected to reach the accuracy of 80.3%, with True Positive (TP) of 82.8% and True Negative (TN) of 57.9%. **D** The graph shows the ROC curve of the model validation on the test set = AUC value of 0.735.

Univariate logistic regression was performed on each of the 109 LRRK2 interactors with significant differential expression in the sPD and/or LRRK2-PD cohorts vs. controls, out of which 11 interactors with *p*-value < 0.05 were selected for further model construction, including TUBB6, SNCA, HSPA1A, BAG3, ACTA2, TUBG1, LMNB1, CDK2, RAB5B, LRRK2 and SLC25A6 (Table S5). A LASSO regression model constructed with these 11 interactors was then trained on a randomly picked cohort of 296 sPD cases (coded as 1) and 80 LRRK2-PD cases (coded as 0). A λ value of 0.006 (log(λ) = −5.062 was chosen to reach the minimum MSE = 0.316 (Fig. 4A), leaving a total of 9 interactors in the model, including CDK2 (beta = −0.069), RAB5B (beta = −0.967), ACTA2 (beta = −0.910), TUBB6 (beta = −1.051), LRRK2 (beta = 0.224), HSPA1A (beta = 3.103), LMNB1 (beta = 0.611), SNCA (beta = −1.027), SLC25A6 (beta = −1.328) (Fig. 4B). The cut-off on the predicted value was optimised as 0.54 to reach the maximum accuracy in the training set of 80.3%, with True Positive (TP) of 82.8% and True Negative (TN) of 57.9% (Fig. 4C). The refined model was then validated on the test set, containing 75 sPD cases and 20 LRRK2-PD cases. ROC curve showed an AUC = 0.735 (95% CI: 0.617–0.853), suggesting a good classification performance of the LASSO model built upon the mRNA levels of LRRK2 interactors (Fig. 4D).

### Co-expression modules of LRRK2 interactors in the sPD and LRRK2-PD conditions

**Fig. 5:**
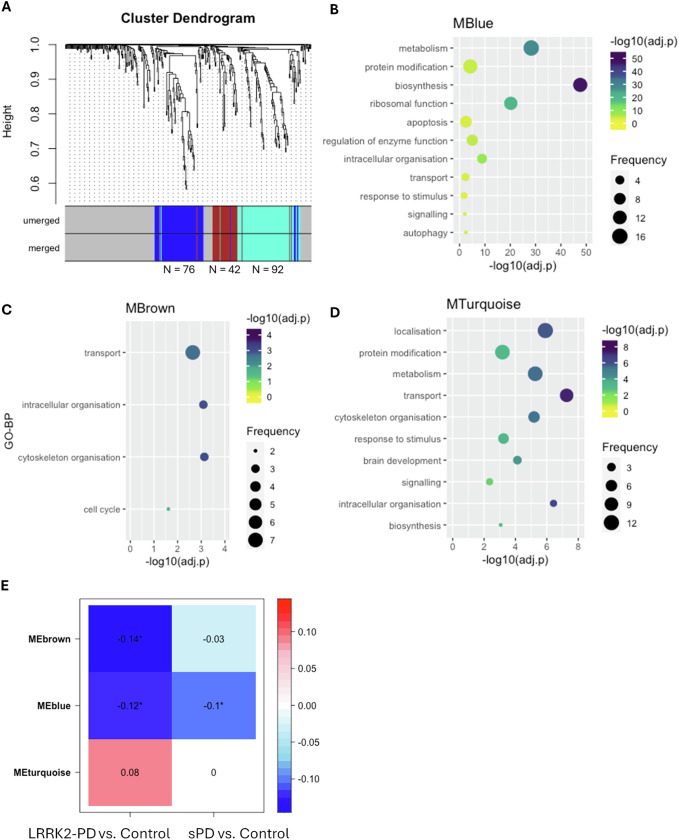
WGCNA on LRRK2 interactors in the sPD, LRRK2-PD and control conditions. **A** The dendrogram shows the 3 co-expression modules identified among LRRK2 interactors across the 3 cohorts. Modules are represented by colours (MBlue, MBrown and MTurquoise). **B**–**D** The bubble plots show the semantic groups of GO-BPs enriched for MBlue, MBrown and MTurquoise. The bubble colour represents the adjusted-p value of the most significant GO-BP within each semantic group (reported on the vertical axis), while the bubble size represents the number of GO-BPs in each semantic group. **E** The heatmap shows the Module-Trait correlation between the eigengene of the 3 co-expression modules (MEblue, MEbrown and MEturquoise) and PD type. The numbers in cells and cell colours represent Pearson’s coefficients. Significant correlation was defined as Pearson’s *p*-value < 0.05 (marked with *).

A signed gene co-expression network was constructed across the 3 examined cohorts via WGCNA with the soft power β = 12 (Fig. S3), within which a total of 3 co-expression modules were identified: MTurquoise (*N* = 92 interactors), MBlue (*N* = 72 interactors) and MBrown (*N* = 42 interactors) (Fig. 5A, Table S6). Of note, LRRK2 was found in none of these 3 modules, suggesting that the overall co-expression level between LRRK2 and its interactors was relatively low in the whole blood, which is in accordance with the findings in our previous study^2^. Functional enrichment analysis associated the MBlue with metabolism (*N* = 16/82 GO-BPs, 19.5%), protein modification (*N* = 13/82 GO-BPs, 15.8%), biosynthesis (*N* = 13/82 GO-BPs, 15.8%), ribosomal function (*N* = 11/82 GO-BPs, 13.4%) and apoptosis (*N* = 8/82 GO-BPs, 9.7%) (Fig. 5B, Table S7); MBrown was enriched for transport (*N* = 7/15 GO-BPs, 46.7%), intracellular organisation (*N* = 3/15 GO-BPs, 20.0%), cytoskeleton organisation (*N* = 3/15 GO-BPs, 20.0%) and cell cycle (*N* = 2/15 GO-BPs, 13.3%) (Fig. 5C, Table S7); while MTurquoise was associated with localisation (*N* = 12/62 GO-BPs, 19.4%), metabolism (11/62 GO-BPs, 17.7%), protein modification (11/62 GO-BPs, 17.7%) and transport (9/62 GO-BPs, 14.5%) (Fig. 5D, Table S7). Module-Trait correlation analysis showed that the eigengene of MBlue (MEblue) was significantly down-regulated in the LRRK2-PD and sPD cases as compared to the control cohort (*p* < 0.05), while MEbrown was significantly down-regulated in the LRRK2-PD cases only. No significant changes were identified for the MEturquoise in the 2 PD conditions vs. controls (Fig. 5E). Interestingly, 58 out of 76 LRRK2 interactors within the blue module were found in the interactome of other PD genes (*SNCA, PRKN, PINK1, GBA1, PARK7, VPS35, ATP13A2 FBXO7, SLC6A3, DNAJC6, DCTN1* and *SYNJ1*; Supplementary Data S1).

### Construction of the LRRK2_net_

**Fig. 6:**
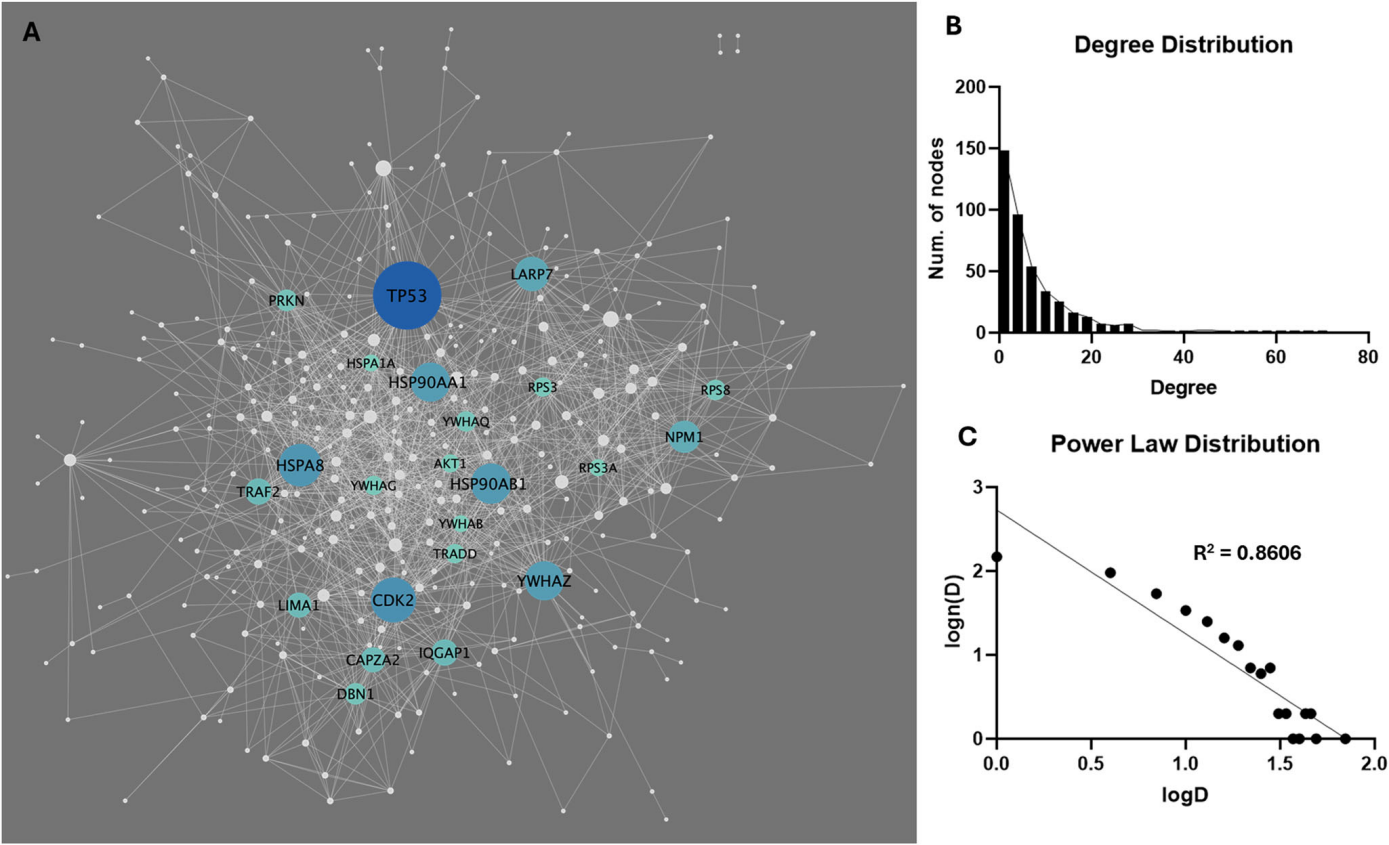
The LRRK2_net_. **A** The network graph shows the LRRK2_net_, in which nodes represent the LRRK2 interactors (*N* = 338), while edges represent the “2-layer” PPIs (*N* = 1345). Node size refers to the node degree. Interactors with higher centrality (with degree ≥ 24) were colour-coded according to their degree. **B** The bar graph shows the distribution of degrees for the LRRK2 interactors. **C** The log–log plot shows that the LRRK2_net_ follows the power law, in which the *X*-axis represents the log-transformed degree (logD), while the Y-axis represents the log-transformed frequency of a LRRK2 interactor with a certain degree level (log(*n*(*D*))). The scatters fit a linear regression line with R-square = 0.8606.

A total of 4860 connections (interactions) across the LRRK2 interactors were extracted from the HIPPIE database (v2.3), among which 1466 (30.2%) were scored as “high confidence” (HIPPIE confidence score ≥ 0.72), out of which 121 self-interactions were removed from the list, thereby leaving 1345 “2^nd^-layer” PPIs for 338 LRRK2 interactors for LRRK2_net_ construction (Fig. 6A, Table S8). Degree (i.e., the number of PPIs connected to a given interactor) distribution analysis showed that 216 interactors (51.7%) had degrees ≤ 4; 141 interactors (33.7%) presented degrees between 5 and 14; 41 interactors (9.8%) presented degrees between 15 and 24; 24 interactors showed degree ≥ 24, suggesting that the LRRK2_net_ follows the Power Law distribution (log–log plot R-square = 0.8606) (Fig. 6B, C, Table S9). Interactors with degree ≥ 24 (the top 5% of all) were defined as “sub seed” proteins in the LRRK2_net_, with TP53 (degree = 68), CDK2 (degree = 48), HSPA8 (degree = 46), HSP90AB1 (degree = 44), HSP90AA1 (degree = 43), YWHAZ (degree = 43), LAPR7 (degree = 39), NPM1 (degree = 37), TRAF2 (degree = 32), IQGAP1 (degree = 32), LIMA1 (degree =31), CAPZA2 (degree = 31), PRKN (degree = 28), DBN1 (degree = 28), YWHAQ (degree = 27), RPS8 (degree = 27), YWHAG (degree = 26), TRADD (degree = 26), RPS3 (degree = 26), AKT1 (degree = 25), YWHAB (degree = 24), HSPA1A (degree = 24), RPS3A (degree = 24) presenting the highest degree, suggesting that these proteins may play an essential role in maintaining the local connectivity of the LRRK2_net_.

### Weighted network analysis on the LRRK2_net_

**Fig. 7:**
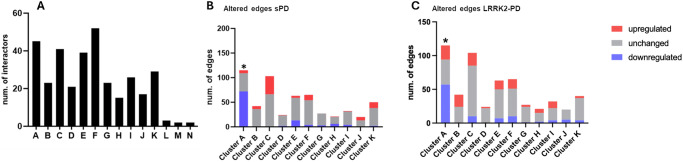
Topological clustering of the LRRK2_net_. **A** The bar graph shows the 14 topological clusters identified in the LRRK2net via the Fast Greedy Algorithm. Cluster L, M, N were discarded from further analysis due to their small size (they contained ≤ 5 interactors). **B** The bar graph shows the impact of expression changes linked to the sPD condition on the edges of each topological cluster. Upregulated edges (in red) were defined as (1) with ≥ 1 connected interactor exhibiting increased expression level in sPD as compared to controls; and/or 2) 2 connected interactors positively co-expressed (with Pearson’s coefficient > 0.6) in sPD but not in controls. Downregulated edges (in blue) were defined in the opposite way: (1) with ≥ 1 connected interactor exhibiting decreased expression level in sPD as compared to controls; and/or (2) 2 connected interactors positively co-expressed (with Pearson’s coefficient > 0.6) in controls but not in sPD. The percentage of upregulated, unchanged and downregulated edges were compared within each cluster via One Sample Proportion test. Only Cluster A was significantly downregulated in sPD (*p* < 0.001, *). **C** Same as B but analysis comparing LRRK2-PD vs. Controls. Cluster A was significantly downregulated in LRRK2-PD (*p* < 0.05, *).

A total of 14 topological clusters were identified in the trimmed-LRRK2_net_ using the Fast Greedy algorithm based on the measure of edge betweenness (Fig. 7A, Table S9). Of note, 3 clusters containing less than 5 interactors each were removed (considering a cut-off threshold on cluster connectivity ≥5 proteins), leaving a total of 11 clusters for further analysis. For each of the 11 topological clusters, edges were classified as up/down-regulated or unchanged bases on the differential expression and co-expression levels of LRRK2 interactors in the sPD and LRRK2-PD conditions as compared to the controls. The distribution of the edges across these 3 categories was compared via One Sample Proportion Test to identified clusters significantly altered in expression in sPD or LRRK2-PD in comparison with controls (Fig. 7B, C).

**Fig. 8:**
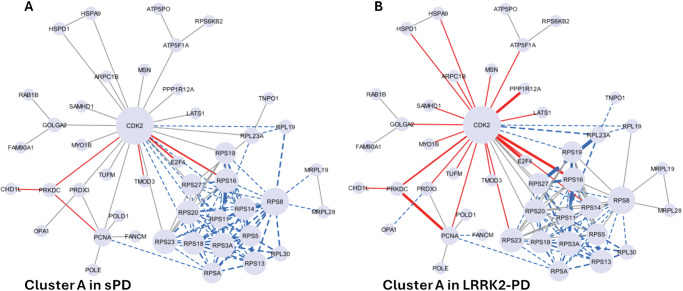
Details of cluster A in LRRK2-PD and sPD. The network graphs show the significant downregulation of Cluster A in sPD (**A**) and LRRK2-PD (**B**), in which LRRK2 interactors are represented as nodes (*N* = 45) while PPIs are represented as edges (*N* = 115). Edges are represented with a continuous red line if they are up-regulated, with a dotted blue line if they are down-regulated. The thickness of the edges refers to the level of alterations of PPIs: the line is thicker if the 2 interactors connected by a given edge exhibited both the same trend of alteration (i.e, both up-regulated or down-regulated in PD cases vs. Controls).

Among the 11 clusters, Cluster A was significantly altered (down-regulated) in both sPD and LRRK2-PD cases vs. controls (*p* < 0.05), with 72/115 (62.6%) and 57/115 (49.5%) edges down-regulated, respectively (Fig. 7B, C, Fig. 8A). Of note, out of the 14 down-regulated interactors, 12 of them were ribosomal proteins. Functional enrichment analysis associated Cluster A with gene translation and ribosomal functions, suggesting that the sPD and LRRK2-PD pathologies potentially contribute to perturbed ribosomal homoeostasis and translation process by down-regulating this cluster of LRRK2 interactors (Fig. 8B, Table S10).

## Discussion

Multifactorial neurodegenerative disorders such as PD present with a complicated aetiopathogenesis, triggered by multiple causative events (or risk factors) from the environment and from the genome. In PD, for example, the majority of the patients have a sporadic form of the disease, with no large effect size genetic variants contributing to etiology; these cases are considered to be due to a complex interplay of small effect size genetic risk factors in combination with a triggering environmental exposure. The often-transient nature of environmental exposures are difficult to study, however PD has been linked to long-term exposure to air pollution and chemicals^33–36^, while old age remains the major risk factor for PD^37,38^. In contrast, a minority of patients present with a familial pattern of disease, with at least one mutation with effect size large enough to drive neurodegeneration. The sporadic and the genetic forms of PD are, therefore, by definition triggered by different combinations of risk factors. This poses the question as to whether, despite the similar clinical presentation and the classification under the same disease name, sporadic and genetic forms might represent a more nuanced spectrum of disorders. This nosological question holds the key to a very practical issue: sporadic disorders are difficult to be modelled in vitro, thus the scientific community frequently relies on genetic models based on the familial forms of the same disease to simulate the disease scenario in vitro and in vivo. These experimental models might not be accurate if we are indeed studying a spectrum disorder where the same clinical manifestation may be triggered by different molecular scenarios. Similarly, a therapeutic approach targeted to the molecular core of the neurodegeneration developed for the genetic forms of the disease might not be fully effective on the sporadic disease, thus requiring cohort-specific interventions. In this study, we applied a systems biology approach to generate a model and investigate the potential molecular differences between sPD and LRRK2-PD, focusing on the transcriptomic expression profile of the LRRK2 protein interactome. We considered that the LRRK2 functionality is orchestrated by the protein interactions that interlink LRRK2 with the cell proteome. It has previously been reported that LRRK2 interaction behaviour is affected by the presence of mutations^39^; we therefore speculated that the presence of PD causing mutations in LRRK2 (LRRK2-PD) would modify the LRRK2 connectivity and in turn trigger expression changes within the LRRK2 interactome. These changes might be specific for the LRRK2-PD scenario since no LRRK2 mutations are present in sPD. However, it is also possible that expression changes of the LRRK2 interactome happen just as a consequence of PD, in a feedback response to the molecular alterations induced by the disease; in this case, these alterations should be evident in both presence (LRRK2-PD) and absence (sPD) of LRRK2 mutations.

There is increasing evidence that the immune system and immune-related functions are deeply linked to the pathogenesis of PD^40^. Indeed, we demonstrate that a large portion of the LRRK2 interactome (37%) is enriched for immune related functions and highly expressed in whole blood in comparison with other peripheral tissues and the CNS. We therefore evaluated expression changes of the LRRK2_int_ (cases vs controls) in whole blood mRNA and found that 28.9% (109/377) of the LRRK2 interactors presented significant changes, among which only 13 showed a similar trend of alteration (4 up-regulated and 9 down-regulated) in both the sPD and LRRK2-PD cases. Among these 109 interactors, 9 were selected by the LASSO regression model differentiating the LRRK2-PD and sPD cohorts including: CDK2, RAB5B, ACTA2, TUBB6, LRRK2, HSPA1A, LMNB1, SNCA, and SLC25A6. This model suggested that, globally, sPD and LRRK2-PD might be differentiated by looking at the transcriptomics profiles of the LRRK2 interactors in whole blood. These findings supported our hypothesis that LRRK2-PD and sPD might be triggered by different molecular alterations and thereby need to be treated as different conditions for biomarker discovery and drug development.

When we carried out a functional analysis of the 109 significantly-altered LRRK2 interactors, we found that proteins up-regulated in the LRRK2-PD condition were mainly related to cytoskeletal dynamics and transport, while those up-regulated in the sPD condition were associated with signalling and protein metabolic processes, again suggesting divergent functional profiles for the LRRK2_int_ in the PD scenario depending on the presence/absence of the LRRK2G2019S mutation. However, the downregulated proteins in both cohorts were associated with metabolic processes and ribosomal assembly suggesting these functions to be consistently altered in both PD scenarios regardless of the presence/absence of the LRRK2-G2019S mutation. Interestingly, the 13 protein whose expression profile was altered in both LRRK2-PD and sPD were similarly related to ribosomal activity and protein biosynthesis, suggesting that there are commonalities at the molecular level between LRRK2-PD and sPD.

The results obtained via DEA were further corroborated by the co-expression analysis. We analysed the co-expression behaviour of the LRRK2 interactome using the classical WGCNA pipeline to identify modules of LRRK2 interactors that are co-express across the sPD, LRRK2-PD and control cohorts. A total of 3 co-expression modules were consistently identified in the 3 conditions, and they might indicate functional units of LRRK2 interactors that participate in communal processes. Module-Trait analyses found that one of the 3 modules (MBlue) was down-regulated in both sPD and LRRK2-PD cases as compared to controls, one (MBrown) was down-regulated only in the LRRK2-PD cohort while the other remained unchanged in both PD cohorts vs. controls. Interestingly, MBlue, altered in both LRRK2-PD and sPD, contained 31 ribosomal proteins (RPs).

All these findings suggested the existence of molecular alterations that are specific to the LRRK2 and sporadic PD conditions, however molecular and functional similarities can also be found. For example, in this in silico investigation, we suggest altered ribosomal functionality and protein biosynthetic processes to be an hallmark of PD, regardless the presence of pathogenic *LRRK2* mutations. In additions, we confirmed that the LRRK2 interactors that are similarly altered in LRRK2-PD and sPD considering DEA and WGCNA are largely represented within the interactome of other PD genes. This observation might suggest their importance to the molecular pathogenesis of PD, regardless the absence or presence of familial mutations.

Similar alterations in protein synthesis/ribosomal functions were indeed observed in previous studies in the blood and substantia nigra tissues of PD patients as well as related animal models^41–44^.

We finally proceeded to identify topological clusters within the LRRK2 interactome, based on the protein connections across LRRK2 interactors. Topological clusters might indicate functional local communities within a larger network based on how proteins relate/connect with each other. The topological clustering algorithm identified 11 clusters in the LRRK2_net_, these are portions of the network that are more connected within each other than the average connection of the entire network. Among these 11 clusters, cluster A presented lower connectivity in both LRRK2-PD and sPD vs controls and this cluster was functionally related to ribosomal functions. The majority of the RP were, as expected, contained within cluster A; this cluster was significantly downregulated, again potentially suggesting that the functionality of RPs and ribosomal/protein biosynthetic processes are universally reduced during PD.

There are a number of limitations to this study: (1) the sample size of the cohorts are relatively small, especially for the LRRK2-PD cohort and larger sample size would improve statistical power and thereby provide more robust results; (2) PD cases recruited by PPMI were at the early stages of the disease and the whole blood mRNA sequencing was run at the first visit; therefore, the alterations of some LRRK2 interactors could be too subtle to be detected by DEA or WGCNA; 3) expression changes (used as proxy for protein levels) in the LRRK2 interactome are dynamic and affected by the local environment (such as absence/presence of inflammation) while (due to the data available) in our analyses protein interactions have been considered as static.

In conclusion, our study suggests that although sPD and LRRK2-PD share defining aspects of neuropathology and clinical characteristics, the molecular pathways underlying the etiology and pathogenesis of the two conditions have important distinct features. There are shared changes of the LRRK2 interactome that can be appreciated at the transcriptome level in both the conditions, mainly associated with alterations of RPs and proteins whose function is important for protein biosynthesis. However, there are also substantial differences between the two conditions suggested by their unique transcriptomics signatures. This conclusion cautions against considering LRRK2-PD and sPD as identical conditions, highlights the need to for specific experimental models to be generated to differentially study sporadic and LRRK2 PD, and confirms the requirement for patient stratification in clinical trials.

## Methods

### LRRK2 protein interactors download and quality control (QC)

LRRK2 protein interactors were downloaded via PINOT v1.1 (http://www.reading.ac.uk/bioinf/PINOT/PINOT_form.html), HIPPIE v2.3 (http://cbdm-01.zdv.uni-mainz.de/~mschaefer/hippie/index.php) and MIST v5.0 (https://fgrtools.hms.harvard.edu/MIST/)^45–47^ on 16^th^ March 2023 and the LRRK2 interactome was built following the pipeline in ref. ^2^. In summary: to access the most comprehensive set of LRRK2 interactors, “Lenient” filter level was applied in PINOT; while no filter was applied for HIPPIE and MIST to download the entire set of raw interaction to be filtered in a second step. Interactors retrieved from the 3 tools were merged and QC-ed to identify interactors with missing publication identifier, missing interaction detection method, no conversion to a standard gene identifier, and with low interaction confidence score.

### LRRK2 protein interactome (LRRK2_int_) in whole blood

In our previous study^2^, we compared the mRNA levels of LRRK2 interactors pair-wise across 11 brain regions and 4 peripheral tissues in healthy individuals derived from the GTEx database (https://www.gtexportal.org/). Tissues were scored based on the pair-wise comparison results for each LRRK2 interactor. Briefly, the higher the score, the higher a certain interactor is expressed in a certain tissue. A specifically high expression level was defined as tissue score ≥ 12, meaning that a given interactor exhibited significantly higher mRNA levels in tissue X as compared to other 12 tissues. For this current study, scores of LRRK2 interactors in the whole blood were extracted from^2^ and the interactors with tissue scores ≥ 12 were analysed via functional enrichment analysis.

### Whole blood RNA-Seq data download and QC

Baseline (BL = time at diagnosis) whole blood mRNA data (read counts) of LRRK2 interactors for healthy controls (HC), sPD patients and LRRK2-PD patients were retrieved using Ensembl gene ID from the Parkinson’s Progression Marker Initiative (PPMI) dataset on 24^th^ March 2023. PPMI is an ongoing observational, international, multicentred cohort study aimed at identifying the biomarkers of PD progression in a large cohort of participants (https://www.ppmi-info.org/). The current PPMI Clinical study protocol (#002) is WCG approved (IRB Tracking #20200597). The previous PPMI Clinical protocol (#001), was IRB reviewed by the University of Rochester Research Subjects Review Board. Informed consent was obtained from all human participants. PPMI is a public-private partnership—is funded by The Michael J. Fox Foundation for Parkinson’s Research and funding partners, including those reported at https://www.ppmi-info.org/about-ppmi/who-we-are/studysponsors. Study protocol and manuals are available online (http://www.ppmi-info.org/study-design). PPMI enroled patients with early, untreated (de novo) Parkinson’s disease as well as healthy controls of similar age and sex as well as genetic cohorts with Parkinson’s disease and non-manifesting carriers of mutations. Pathogenic variants used to define genetic PD cases included 7 PD-related genes, namely *LRRK2, GBA1, VPS35, SNCA, PRKN, PARK7* and *PINK1*.

In this study, we included the “de novo”, “genetic (with LRRK2 mutations)” and “healthy control” cohorts. Subjects from the 3 cohorts were further filtered to keep only those with robust genetic status records using the following criteria: confirmed by at least 3 out of 6 detection techniques (WGS, WES, RNA-Seq, GWAS, CLIA, SANGER) of which 1 should be a next generation sequencing technique (WGS, WES, RNA-Seq) and 1 should be a screening technique (GWAS, CLIA, SANGER). For the healthy control cohort, subjects with pathogenic variants in the above-mentioned PD-related genes were excluded. PD patients with no pathogenic variants were defined as the sporadic PD (sPD) cohort, while those with pathogenic variants in the *LRRK2* gene only were defined as the LRRK2-PD cohort. Principal Component Analysis (PCA) was performed on mRNA read counts to remove potential outliers. Metadata of QC-ed subjects at BL were derived from the PPMI database, including gender, age at screen, motor symptom severity (as evaluated by the MDS-Unified Parkinson’s Disease Rating Scale IIII (MDS-UPDRS III) and *LRRK2* mutation type (for the LRRK2-PD cases only). MDS-UPDRS III scores of the sPD and LRRK2-PD cohort were compared via t-test. Transcripts of LRRK2 interactors with read counts ≤ 15 in more than 75% QC-ed subjects were removed^48^. Read counts of LRRK2 interactors retrieved from PPMI were extracted for the 3 cohorts, thereby forming the “PPMI_Matrix”.

### Differential Expression Analysis (DEA) and classification models for sPD and LRRK2-PD

The PPMI_Matrix was then normalised via the median of ratios method using the “count” function in the R package “DESeq2”^49^. The normalised PPMI_Matrix (hereby referred as “norm_PPMI_Matrix”) was utilised to perform DEA to compare the expression levels of LRRK2 interactors in the control, sPD and LRRK2-PD cohorts using “DESeq2” and calculating fold change (FC) for each of the LRRK2 interactors (*i*) in [sPD vs control] and [LRRK2-PD vs control]. P-value adjustment for multiple comparisons were performed via Bonferroni’s method. Of note, results from DEA were adjusted for sex. LRRK2 interactors were considered significantly altered when |log2FC| > 0.05 and adjusted-*p* < 0.05 in [sPD vs control] or [LRRK2-PD vs control]^50^. Up/down-regulated LRRK2 interactors in the 2 PD conditions were functionally annotated via Gene Ontology Biological Process (GO-BP) enrichment analysis. The read counts of LRRK2 interactors with significant alterations in the 2 PD conditions as compared to controls were utilised to construct a machine learning model via Least Absolute Shrinkage and Selection Operator (LASSO) algorithm using the R package “glmnet”. Of note, in order to reduce the risk for model overfitting, univariate logistic regression was performed on each LRRK2 interactor prior to model training and only those with p-value < 0.05 were included in the LASSO regression model. The train-test split ratio for the LASSO regression model was set as 4:1. The tunning parameter lambda (λ) were optimised by a 10-fold cross-validation (CV) to reach the minimum Mean-Squared Error (MSE) via the “cv.glmnet” function of the “glmnet” package. The refined models were then assessed on the test set. Receiver Operating Characteristic (ROC) curves were generated via the “roc.glmnet” function of the “glmnet” package.

### Weighted Gene Co-expression Network Analysis (WGCNA)

Signed Weighted Gene Co-expression Network Analyses (WGCNA) were performed on the norm_PPMI_Matrix via the R package “WGCNA” to identify co-expression modules within the LRRK2_net_ across the sPD, LRRK2-PD and control conditions. Module-Trait correlation was evaluated via the “corPvalueStudent” function in the “WGCNA” package.

### LRRK2 PPI network (LRRK2_net_) construction and weighted network analysis

To construct the LRRK2_net_, the 2^nd^-layer PPIs (i.e., PPIs among LRRK2 interactors) were downloaded via HIPPIE (v2.3) on 16^th^ March 2023. The 2^nd^-layer PPIs with high confidence score (≥ 0.72) were kept for network construction (the LRRK2_net_). The Fast Greedy Clustering algorithm^51^ was utilised to detect topological clusters in the LRRK2_net_ based on edge betweenness (i.e., calculating the number of shortest paths between any pair of nodes in the network that pass-through a given edge), via the “cluastermaker2” Cytoscape add-in (v2.3.4). For each obtained topological cluster, edges were classified as up/down-regulated or unchanged based on the following criteria: A) up-regulated edge: i) at least 1 of the 2 nodes connected by the edge had increased expression level in sPD and/or LRRK2-PD vs controls or ii) a strong positive co-expression (Pearson’s coefficient > 0.6) was observed for the 2 nodes connected via the edge in sPD and/or LRRK2-PD but not in controls; B) downregulated edge: i) at least 1 of the 2 nodes connected by the edge presented decreased expression level in sPD and/or LRRK2-PD vs controls or ii) a strong positive co-expression (Pearson’s coefficient > 0.6) for the 2 nodes connected via the edge was observed in the controls but not in sPD and/or LRRK2-PD cases. The percentage of upregulated, downregulated and unchanged edges for each single topological cluster were calculated for the sPD and the LRRK2-PD scenarios and compared via One Sample Proportion Test to identify the trend of each topological cluster and qualitatively define whether a cluster was mainly up/down regulated or unchanged in sPD or LRRK2-PD vs controls.

### Functional enrichment analysis

In this study, functional enrichment analyses for LRRK2 interactors were performed via the webtool “g:Profiler” (https://biit.cs.ut.ee/gprofiler/gost)^52^. The parameters were set as follows: organism—Homo sapiens (Human); data source—GO biological process (GO-BPs) only; statistical domain scope—annotated genes only; statistical method—Fisher’s one-tailed test; significance threshold—Bonferroni correction (threshold = 0.05). No hierarchical filtering was included. To increase the sensitivity of analysis, a cut-off of ≤ 2500 was set for the “term size” of enriched GO terms. For larger GO term lists, GO-BPs were grouped based on semantic similarity and text cloud analysis was performed to extract keywords from term names via the R package “wordcloud” (https://CRAN.R-project.org/package=wordcloud).

### Supplementary information

Supplementary Figures and Data

Supplementary Tables

## Data Availability

Data used in the preparation of this article were obtained from the Parkinson’s Progression Markers Initiative (PPMI) database (www.ppmi-info.org/access-data-specimens/download-data), RRID:SCR_006431. For up-to-date information on the study, visit www.ppmi-info.org.

## Code availability

The pipelines used in this work are based on publicly available R packages as detailed in the methods section.
