# Supplementary material for: Transcriptomics and weighted protein network analyses of the LRRK2 protein interactome reveal distinct molecular signatures for sporadic and LRRK2 Parkinson’s Disease
Source: NPJ Parkinsons Dis. 2024 Aug 3;10:144. doi: 10.1038/s41531-024-00761-8 (PMC11297940; doi:10.1038/s41531-024-00761-8)
Supplement: Supplementary file 1 — Supplementary Figures and Data [file 41531_2024_761_MOESM1_ESM.pdf]

Figure S1

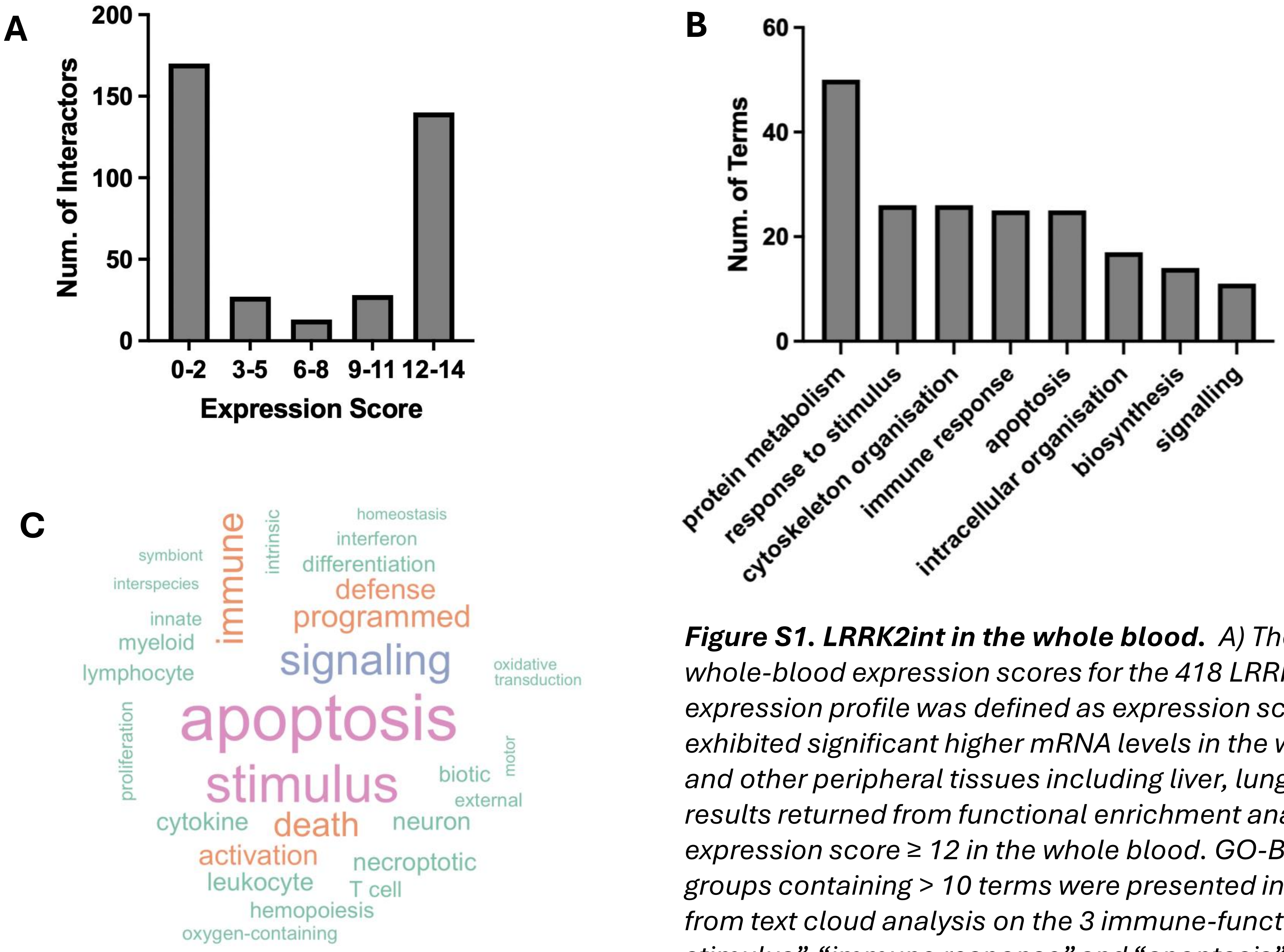

Figure S1. LRRK2int in the whole blood. *A) The bar graph shows the distribution of whole-blood expression scores for the 418 LRRK2 interactors. A significant high expression profile was defined as expression score ≥ 12, meaning these LRRK2 interactors exhibited significant higher mRNA levels in the whole blood as compared to brain regions and other peripheral tissues including liver, lung and kidney; B) The bar graphs showes the results returned from functional enrichment analysis on LRRK2 interactors with expression score ≥ 12 in the whole blood. GO-BP terms were semantically grouped. Only groups containing > 10 terms were presented in the graph; C) The graph shows the results from text cloud analysis on the 3 immune-function-related GO-BP groups: "response to stimulus", "immune response" and "apoptosis".*

Figure S2

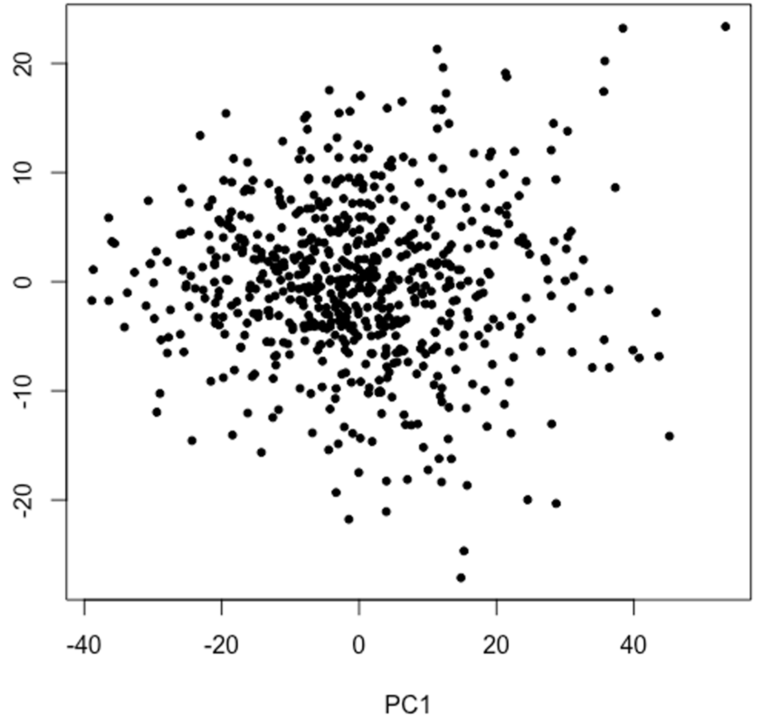

ure S1. Subject QC on PPMI cohort *PCA was performed on the matrix of whole*
od mRNA levels of the LRRK2 interactors for the 3 PPMI cohorts (control, sPD and
RK2-PD). No outliers were excluded from further analysis

Figure S3

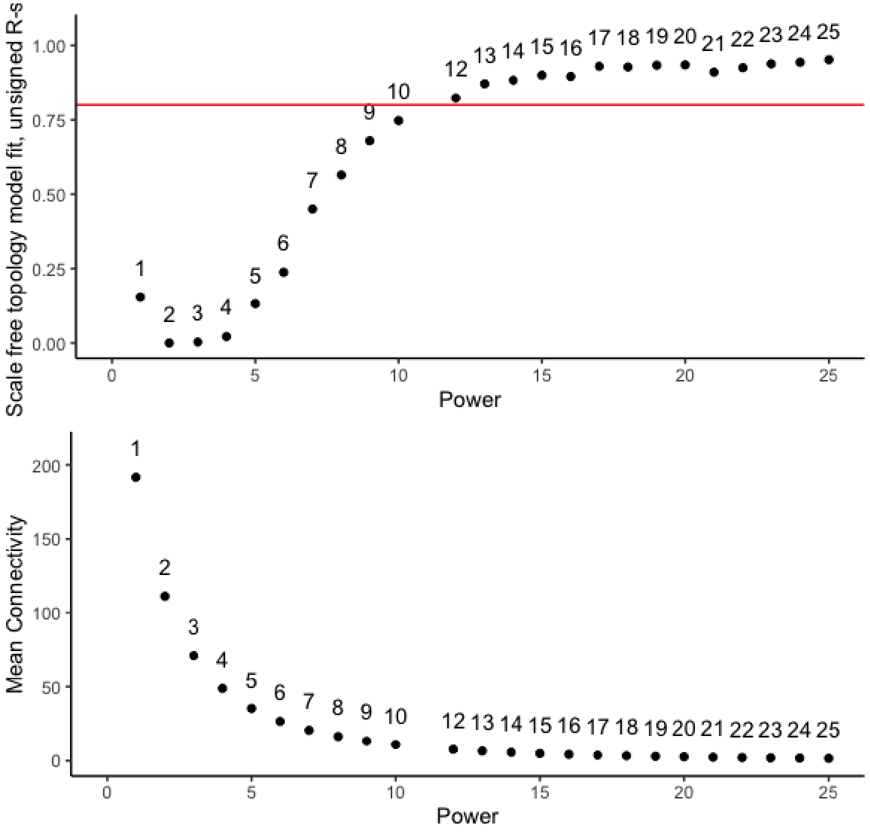

Figure S3. Soft power selection for WGCNA. *The scatter plots shows the selection of aA soft power (β = 28) was selected for the signed co-expression network constructed among LRRK2 interactors across the 3 cohorts, which achieved scale free model fit and a low mean connectivity.*

Data S1

Two resources were used to compile a catalogue of genes associated with Mendelian Parkinson's Disease (PD). The first resource, the International Parkinson and Movement Society (MDS gene) database accessible at https://www.mdsgene.org/g4d, and the second, the NHS Genomic Medicine Service (GMS) Panels Resource, retrievable from https://panelapp.genomicsengland.co.uk/panels/, were utilized for this purpose. Within the MDS gene database, terms categorized under "X-linked dystonia-parkinsonism," "Other forms of dystonia-parkinsonism," "Rapid-onset dystonia-parkinsonism," and Parkinsonism (PARK) were selected, accounting for a total of 17 terms. Within the NHS GMS Panels Resource, a search was conducted for "Parkinson Disease and Complex Parkinsonism" (Version 1.121) panels, focusing on entities labeled "Green," resulting in 43 terms. The intersection of these two sets of extracted terms yielded 12 common genes, which were subsequently manually validated with the addition of "*GBA*". Consequently, the final list comprised 13 genes: *ATP13A2, DCTN1*, *DNAJC6*, *FBXO7, GBA*, *PARK7*, *PINK1*, *PRKN, SLC30A10, SLC6A3, SNCA*, *SYNJ1*, and *VPS35*.
Subsequently, direct protein-protein interactors for each of the chosen PD genes were retrieved from 3 databases: PINOT, HIPPIE, and MIST.
Overlaps between the PD genes interactors and LRRK2 interactors (altered in both sPD and LRRK2 PD in the DEA and WGCNA analyses) were identified (Figure A and Table A and B).

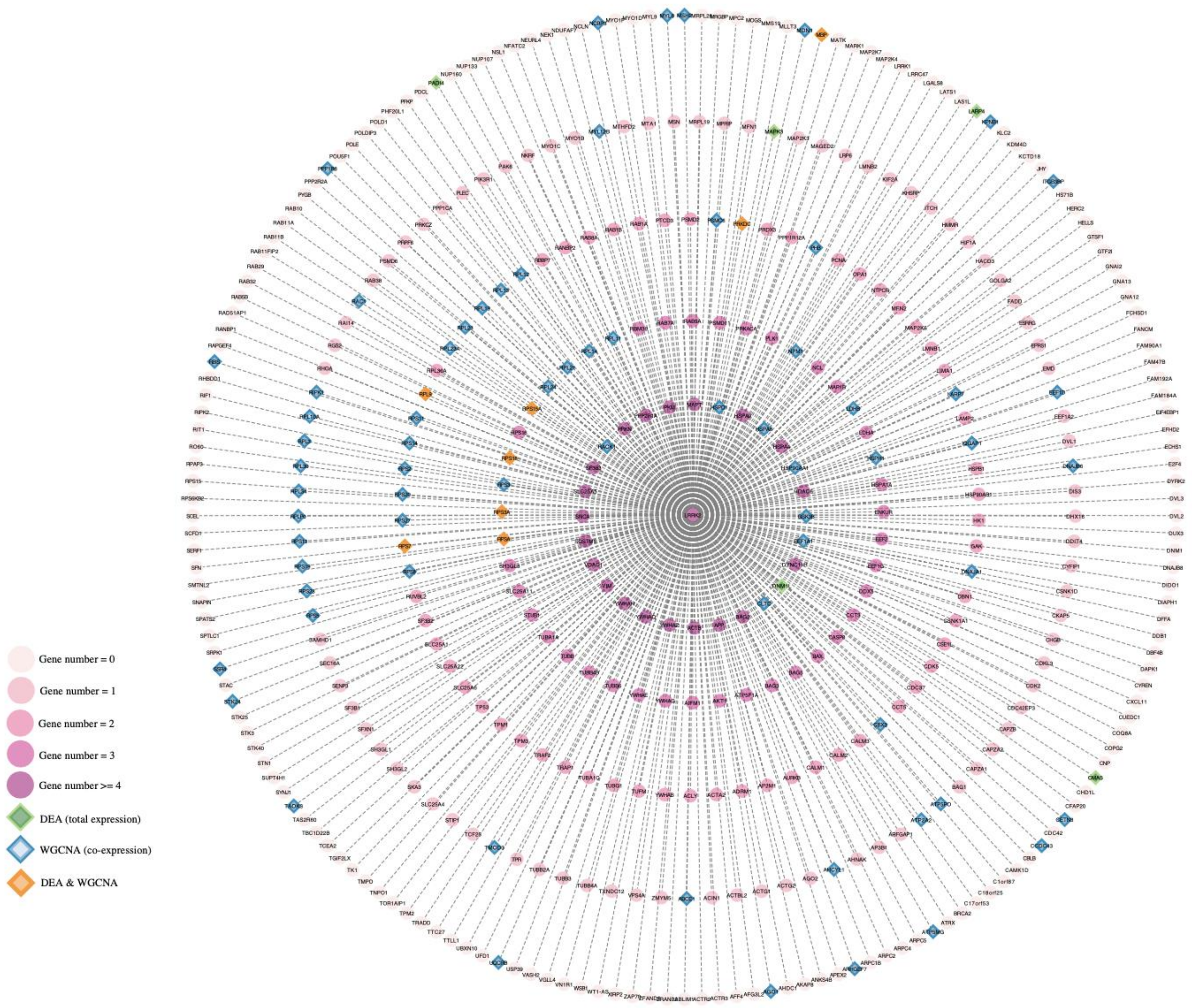

Figure A (Data S1) - The LRRK2 interactome. Each node represents an interactor of LRRK2. The node color corresponds to the number of other Mendelian PD genes interacting with each LRRK2 interactor; with the arrangement of nodes from the outer (0 common interactions) to the inner circle (>4 common interactions).

Additionally, diamond-shaped nodes denote distinct types of alterations in the expression of LRRK2 interactors in both sPD and LRRK2-PD conditions: green diamonds indicate total expression changes (DEA), blue diamonds denote co-expression alterations (WGCNA), and orange diamonds identifies alterations in both DEA&WGCNA.

Table A (Data S1) - Overlaps between the PD genes interactors and LRRK2 interactors altered in DEA

SNCA	PRKN	PINK1	GBA	DJ-1	VPS35	ATP13A2	FBXO7	SLC30A10	SLC6A3	DNAJC6	DCTN1	SYNJ1
MAPK3	DNM1L	DNM1L		DNM1L	DNM1L		RPS15A					
	PRKDC	PRKDC					RPS18					
	RPL9	RPL9					RPS3A					
	RPS15A	RPS15A					RPSA					
	RPS18	RPS18										
	RPS3A	RPS3A										
	RPS7	RPS7										
	RPSA	RPSA										

Table B (Data S1) - Overlaps between the PD genes interactors and LRRK2 interactors altered in WGCNA

SNCA	PRKN	PINK1	GBA	DJ-1	VPS35	ATP13A2	FBXO7	SLC30A10	SLC6A3	DNAJC6	DCTN1	SYNJ1
CLTC	PRKDC	PRKDC	HSP90AA1	IQGAP1	CBX3	HSPA8	RPS15A		RACK1	CLTC	CLTC	CLTC
EEF1A1	RPL9	RPL9	HSPD1	LDHB	GSK3B	CLTC	RPS18			AHCYL1	EEF1A1	
GSK3B	RPS15A	RPS15A		NPM1	PHB	HSPH1	RPS3A				LARP7	
HSP90AA1	RPS18	RPS18		RACK1	RPL24		RPSA				GSK3B	
HSPA8	RPS3A	RPS3A		TMOD3			CLTC					
HSPH1	RPS7	RPS7					DNAJA1					
	RPSA	RPSA					DNAJB6					
	ABCE1	ATP2A2					EEF1D					
	ATP5PO	CLTC					GSK3B					
	CBX3	EEF1A1					HSP90AA1					
	DNAJA1	HSP90AA1					HSPD1					
	EEF1A1	HSPA8					RACK1					
	HSP90AA1	HSPD1					RPL11					
	HSPA8	IQGAP1					RPL14					
	HSPD1	LARP7					RPL21					
	HSPH1	LDHB					RPL23					
	LDHB	NPM1					RPS11					
	MYL12B	PSMC6					RPS2					
	NPM1	RACK1					RPS20					
	PHB	RPL11					RPS27					
	PSMC6	RPL12					RPS3					
	RAC1	RPL13										
	RACK1	RPL14										
	RIPK1	RPL19										
	RPL10A	RPL21										
	RPL11	RPL23A										
	RPL12	RPL24										
	RPL13	RPS14										
	RPL14	RPS3										
	RPL19	RPS8										
	RPL21											
	RPL23											
	RPL23A											
	RPL24											
	RPL3											
	RPL30											
	RPL34											
	RPLP0											
	RPS11											
	RPS13											
	RPS14											
	RPS19											
	RPS2											
	RPS20											
	RPS23											
	RPS27											
	RPS3											
	RPS5											
	RPS8											
